# Diagnostic discrepancies between emergency department admissions and hospital discharges among older adults: secondary analysis on a population-based survey

**DOI:** 10.1590/1516-3180.0471.R1.05032020

**Published:** 2020-09-11

**Authors:** Thiago Junqueira Avelino-Silva, Michael Alan Steinman

**Affiliations:** I MD, PhD. Physician and Adjunct Professor, Division of Geriatrics, Department of Internal Medicine, Hospital das Clinicas (HC), Faculdade de Medicina FMUSP, Universidade de São Paulo; Vice-Director, Laboratorio de Investigacao Medica em Envelhecimento (LIM-66), Division of Geriatrics, Hospital das Clinicas, Faculdade de Medicina FMUSP, Universidade de São Paulo, São Paulo (SP), Brazil.; II MD. Physician and Professor of Medicine, Division of Geriatrics, Department of Medicine, University of California San Francisco (UCSF), San Francisco (CA), United States; Professor of Medicine, San Francisco Veteran Affairs Medical Center, San Francisco (CA), United States.

**Keywords:** Aged, Diagnosis, Emergency medicine, Hospital medicine, Diagnostic errors, Patient safety, NHAMCS, Atypical presentation, Clinical assessment

## Abstract

**BACKGROUND:**

Older adults frequently experience nonspecific clinical features. However, there is limited evidence on how often admission diagnoses for hospitalized older patients are incorrect, potentially leading to treatment delays.

**OBJECTIVES:**

To determine the consistency between hospital admission and discharge diagnoses, and identify factors associated with diagnostic discrepancies in older adults.

**DESIGN AND SETTING:**

Population-based cohort study in the United States. We included adults aged ≥ 18 years who were admitted from emergency departments (EDs) to hospitals, identified using the 2005-2010 National Hospital Ambulatory Medical Survey, a nationally representative survey.

**METHODS:**

Three admission diagnoses and the principal discharge diagnosis were captured and classified as discrepant if they involved considerably different conditions within the same organ system, or different organ systems altogether.

**RESULTS:**

Each year, 12 million adults were hospitalized following ED visits in the United States; 45% were aged ≥ 65 years. These patients' mean age was 79 years and 58% were women. Diagnostic discrepancies between admission and discharge were more common among adults ≥ 65 years (12.5 versus 8.3%; P < 0.001). Certain admission diagnoses had particularly high rates of diagnostic discrepancies: 26-27% of patients presenting with mental disorders or with endocrine and metabolic diseases had substantial diagnostic discrepancies between admission and discharge. Substantial diagnostic discrepancy was independently associated with longer hospitalization and higher in-hospital mortality.

**CONCLUSION:**

One out of eight older adults hospitalized from EDs was discharged with a principal diagnosis differing considerably from the admission diagnosis. Given that missed or delayed diagnoses are a critical safety problem, clinicians should be vigilant and frequently cogitate alternative diagnostic possibilities.

## INTRODUCTION

Emergency department (ED) visits by patients aged 65 and older have steadily increased over the past decade.^1^ Moreover, there has been a substantial increase in the intensity of resource utilization among older adults, including hospital admissions, intensive care unit admissions, return visits and readmissions, and use of advanced imaging and laboratory tests.^1^^,^^2^ This is explained not only by the aging of the population, but also by the fact that older adults often have complex conditions aggravated by multiple comorbidities, polypharmacy, functional impairment and cognitive decline.^3^^-^^6^

Diagnosis and treatment are further complicated because such patients might present atypical signs and symptoms of disease, thus increasing the degree of clinical uncertainty involving their cases.^3^^,^^7^ Emergency physicians have reported feeling inadequately trained to address geriatric issues and having greater difficulty when managing older adults with diverse clinical presentations.^8^^,^^9^ Previous research findings indicate that older adults are at higher risk of missed diagnoses.^2^ In a study including 103 individuals aged 65 years or more, conducted by Caterino et al., up to 18% of older patients diagnosed with infection during an ED stay were not subsequently diagnosed as infected after admission.^10^ Likewise, Thomas et al. observed in a cohort of 102 elderly subjects that at least a third of the patients clinically diagnosed with dehydration at admission were not dehydrated.^11^

Despite such concerns, these previous studies have generally focused on misdiagnosis of specific clinical conditions. Given that they came from single hospitals or limited samples, their generalizability is uncertain. In summary, there is limited knowledge about how diagnostic uncertainty affects older patients' care, or how often the admission diagnosis for hospitalized older adults turns out to be incorrect.^12^ This is an important gap because missed or delayed diagnoses might give rise to major risks regarding patient safety. A patient's diagnosis at the time of admission prompts the initial course of treatment and, if inaccurate, may lead to wasting valuable time on unnecessary measures, while critical ones are neglected.^13^ Understanding the broader epidemiology of this phenomenon and identifying its risk factors could raise the awareness of front-line healthcare professionals regarding the complexities of caring for older adults and might consequently improve their ability to provide timely and adequate treatment.

## OBJECTIVES

We used nationally representative data from the United States to better understand diagnostic discrepancies in older adults, through evaluating the incidence of substantial diagnostic discrepancies between hospital admission and discharge among these patients, compared with subjects aged 18 to 64. We further aimed to identify potential risk factors associated with occurrences of diagnostic disagreements relating to older adults, and to assess whether such events were associated with unfavorable outcomes in this population.

## METHODS

### Study design and population

This was a secondary analysis on data collected from the 2005-2010 National Hospital Ambulatory Medical Survey (NHAMCS). NHAMCS data are annually collected and generated through a multistage sample design that provides a national probability sample of ED visits made to non-federal, general and short-stay hospitals.^14^ It identifies primary sampling units across the country, then sampling hospitals within each primary sampling unit and, finally, visits to these locations' emergency services. Trained hospital employees abstract the data using standardized entry forms, which varied little from 2005 to 2010.

The 2005-2010 NHAMCS datasets include information from 208,956 ED records representing 740 million encounters. We analyzed the incidence of diagnostic discrepancies among adults aged 18 or over who were admitted from EDs to hospitals and restricted our detailed analysis of risk factors to the population of adults aged 65 years or more. We excluded ED visits that did not lead to hospitalization, ED deaths and patients younger than 18 years of age.

### Study protocol and definitions

The NHAMCS database contains fields for up to three ED physicians' diagnoses (one primary and two secondary) and for one principal hospital discharge diagnosis, which are coded in accordance with the International Classification of Diseases, ninth revision, clinical modification (ICD-9-CM). Diagnoses are abstracted from information on medical records (not from billing information).

### Exclusion of nonspecific diagnoses

We compared the diagnoses at hospital admission and hospital discharge with the goal of identifying diagnostic discrepancies between reasonably specific admission and discharge diagnosis. For this reason, we sought to remove from consideration diagnoses that were nonspecific (e.g. “chest pain”, “altered mental status” or “cough”), since these often represent diagnostic uncertainty and are more difficult to interpret. Similarly, we sought to exclude conditions captured in ICD-9 codes that were not truly disease diagnoses (e.g. “abnormal blood chemistry” or “psychiatric examination”). In order to accomplish these exclusions in a systematic manner, ICD-9-CM codes were independently reviewed and classified in accordance with the following categories: (a) diseases and disease processes; (b) organ system-specific symptoms and signs; (c) general/nonspecific symptoms and signs; or (d) test results and procedures. A blinded, experienced third evaluator acted as adjudicator when necessary. Cases in which all the admission diagnoses or the discharge diagnosis were classified as (c) general/unspecific symptoms and signs or (d) test results and procedures were excluded from the analysis, as were records in which diagnostic information was missing from all admission fields or the discharge field (Table 1).

**Table 1:** Examples of how the diagnoses were classified

Admission diagnostic category 1	Admission diagnostic category 2	Admission diagnostic category 3	Discharge diagnostic category	Classification	Justification
Chest pain	Respiratory abnormality	Pulmonary congestion	Chest pain	**Excluded**	All diagnoses are non-specific
Ulcer of lower limb	Cellulitis of leg	Brain injury	Cellulitis of leg	**No diagnostic change**	Same diagnosis
Bipolar disorder	Lack of housing	Depressive disorder	Schizoaffective disorder	**Change to closely related diagnostic category**	Similar disease process, same organ system
Intermediate coronary syndrome	Thrombosis of lower extremities	-	Esophageal reflux	**Change to distantly related diagnostic category**	Different disease process, reasonable differential diagnosis
Closed fracture of patella	Head injury	Pneumonia	Cerebral infarction	**Change to unrelated diagnostic category**	Different disease process, different organ system

### Identification of diagnostic discrepancies

**Figure 1:**
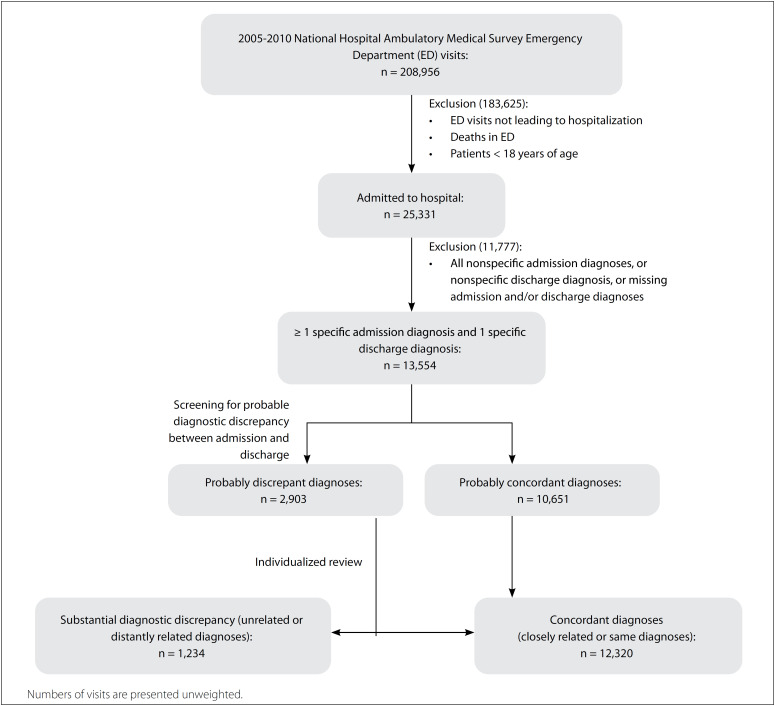
Study design flowchart.

We used an automated two-stage process followed by individualized review of the cases, to identify diagnostic discrepancies (Figure 1). Firstly, we classified the diagnoses using the multilevel diagnoses of the clinical classification software for ICD-9-CM.^15^ This software categorizes ICD-9-CM codes into clinically relevant groups. It aggregates diagnoses following a hierarchical coding system based on the type of disease, in which the top level generally corresponds to an organ system (e.g. diseases of the digestive system), the second level corresponds to broad types of disease within that organ system (e.g. intestinal infection or upper gastrointestinal disorders) and the third and fourth levels correspond to more specific disease states.

We defined admission and discharge diagnoses as probably discrepant when the discharge diagnosis did not match any of the admission diagnoses at the second level of the clinical classification software coding system, i.e. the discharge diagnosis was not within the same broad class of diseases within an organ system as any of the admission diagnoses.

Probable diagnostic discrepancies identified based on the second level of the clinical classification software could still represent ICD-9-CM diagnoses that were in reality similar and not grouped together due to coding inconsistencies. Accordingly, the cases were individually assessed and classified by an experienced clinician using a modified version of the Rating Scale for Diagnostic Change,^16^ which is an instrument designed to classify the degree of concordance between diagnoses over time. By comparing the discharge diagnosis with the closest matching specific admission diagnosis, we classified the diagnosis as follows (Table 1): (1) no category change (the discharge diagnosis referred to the same disease process, with alterations only in wording or specificity); (2) change to a closely related diagnostic category (the discharge diagnosis referred to a different disease process in the same organ, or to a similar disease process in the same system); (3) change to a distantly related category (the discharge diagnosis referred to a different disease process but considered a reasonable differential diagnosis); or (4) change to an unrelated category (the discharge diagnosis referred to a different disease process and different organ system, in comparison with the admission diagnosis). We defined “substantial diagnostic discrepancy” as present when the admission and discharge diagnoses were classified as distantly related (category 3) or unrelated (category 4), or absent in other situations. A second experienced investigator independently reviewed and classified a sample of the cases to assess inter-rater agreement for substantial diagnostic discrepancy, which was 92%. The authors were blinded to all remaining covariates when completing this task.

### Predictors and outcomes of significant diagnostic discrepancy

We evaluated potential risk factors for substantial diagnostic discrepancy among patients aged 65 and over, including age, sex, race/ethnicity, nursing home residence, triage acuity, ED visit in the last 72 hours, hospital discharge within past seven days, presenting diagnosis, pain level, vital signs, level of consciousness at admission, number of ED diagnostic and screening tests, ED length of visit, and length of hospital stay, each of which were recorded on the NHAMCS survey form. Lastly, we investigated the association between substantial diagnostic discrepancy and in-hospital mortality among these patients.

### Statistical analysis

We used the NHAMCS recommended procedures to adjust for the complex survey design and weight the sample to generate nationally representative estimates, with 95% confidence intervals based on standard errors also specified by NHAMCS.^17^ A descriptive analysis was performed using estimated means and proportions, and groups were compared using adjusted Wald tests or designed-based F tests as applicable for continuous or categorical variables, respectively. A multivariate analysis was accomplished using backwards-stepwise logistic regression, including demographic and clinical variables that were statistically associated with substantial diagnostic disagreement in the univariate analysis (P < 0.1) and/or were hypothesized to be conceptually relevant to the model. Missing data were handled using multiple imputation methods. The statistical analyses were performed using Stata 13.1 (Stata Corp, College Station, TX, United States).

Because the datasets are publicly available and contain no patient identifiers, the study was approved as exempt by the institutional review board of the University of California San Francisco and the San Francisco Veteran Affairs Medical Center, on November 15, 2014 (study 14-15031; reference 122992).

## RESULTS

The 2005-2010 NHAMCS datasets included 208,956 unique records, representing an estimated 740 million ED visits in the United States. An estimated 72 million adults were admitted from EDs, of whom 45% were aged 65 years or more. The mean age in this group was 79 years and 58% were women (Table 2). Approximately 19% of the visits by hospitalized patients in all age groups were excluded because of missing diagnostic information, and an additional 27% were excluded due to non-specific diagnostic codes. The proportions of missing and nonspecific diagnoses did not differ between age groups (respectively, P = 0.21 and P = 0.78).

**Table 2:** Older adults' characteristics, and factors associated with substantial diagnostic discrepancy between admission and discharge diagnoses, National Hospital Ambulatory Medical Survey (NHAMCS), 2005-2010 (n = 5,767)^a^

			Weighted proportion, %	Substantial diagnostic discrepancy, %	Adjusted odds ratio	(95% CI)
**Demographic data**						
	**Age (years)**				1.02^e^	(1.01-1.04)
		65-74	35	10		
		75-84	40	13	1.37	(1.05-1.78)
		85 or more	25	15	1.53	(1.16-2.01)
	**Female**		58	12	0.94	(0.76-1.17)
	**Non-Hispanic/Latino**		93	13	0.99	(0.68-1.43)
	**Race**					
		White	84	12		
		Black	12	12	0.98	(0.72-1.37)
		Other	4	14	0.99	(0.67-1.43)
	**Nursing home resident** b		15	18	1.37	(1.04-1.81)
**Clinical data**						
	**Discharged from hospital within last 7 days** d		5	15	1.23	(0.78-1.94)
		Triage acuity^c^				
		Emergency	35	13	1.23	(0.85-1.76)
		Urgent	51	12	1.15	(0.83-1.59)
	**Semi-urgent/ Non-urgent**		14	11		
	**Altered level of consciousness** d		9	17	1.32	(1.02-1.88)
	**Pulse < 60 or ≥ 100 beats/min** b		30	13	1.03	(0.81-1.31)
	**Respiratory rate ≥ 20 insp/min** b		35	12	0.89	(0.69-1.16)
	**Mean arterial pressure < 90 mmHg** b		37	15	1.32	(1.07-1.62)
	**Temperature < 97 °F or ≥ 101 °F** b		17	15	1.13	(0.86-1.49)
	**Number of diagnostic/ screening tests in ED ≥ 7** b		63	13	1.04^e^	(1.01-1.07)
	**Hospital stay ≥ 7 days** b		30	16	1.03^e^	(1.01-1.04)
	**Death** b		3			
**Presenting diagnosis**						
	Diseases of the circulatory system		30	11	0.9	(0.7-1.1)
	Diseases of the respiratory system		20	10	0.7	(0.5-0.9)
	Diseases of the digestive system		13	10	0.8	(0.6-1.1)
	Injury and poisoning		12	7	0.5	(0.3-0.8)
	Diseases of the genitourinary system		8	20	1.7	(1.2-2.3)
	Infectious and parasitic diseases		3	22	1.5	(0.9-2.5)
	Mental illness		3	27	2.8	(1.6-4.7)
	Diseases of the skin and subcutaneous tissue		3	18	1.4	(0.8-2.4)
	Neoplasms		2	17	1.5	(0.8-2.9)
	Endocrine, nutritional and metabolic diseases		2	26	2.9	(1.9-4.5)
	Diseases of the musculoskeletal system and connective tissue		2	20	2	(1.1-3.6)
	Diseases of the blood/ blood-forming organs		1	20	1.8	(0.6-4.9)
	Diseases of the nervous system/ sense organs		1	12	1	(0.4-2.6)

95% CI = 95% confidence interval; ED = emergency department.

a Because adjusting for the complex survey design makes raw numbers (N) not directly proportional to weighted percentages, we present only weighted percentages; Data missing/ unknown:

b < 10%;

c 10%-19%;

d ≥ 20%;

e Per each additional unit (year; test; day).

**Figure 2:**
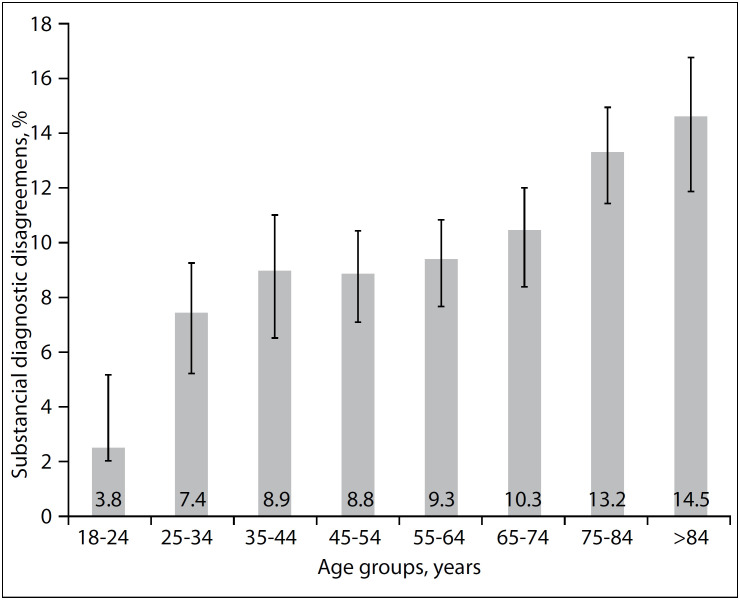
Weighted proportion of visits with substantial diagnostic discrepancy, according to age groups.

Among all the adults included, 10.2% presented substantial diagnostic discrepancy from admission to discharge. The rate of substantial diagnostic discrepancy increased with advancing age (Figure 2). Overall, 12.5% of the patients aged 65 years or older had substantial diagnostic discrepancy versus 8.3% of those aged 18 to 64 (P < 0.001). The subsequent analyses focus on the older age group.

The relationship between demographic and clinical characteristics and having substantial diagnostic discrepancy is shown in Table 2. Patients were more likely to have substantial diagnostic discrepancy when the admission diagnoses referred to diseases of the genitourinary system, mental illnesses, endocrine and metabolic diseases, or diseases of the musculoskeletal system. Patients admitted with diseases of the respiratory system or injuries were less likely to have discrepancies. The three most frequent admission diagnoses that were discrepant, compared with the discharge diagnosis, were urinary tract infection (10% of all discrepant admission diagnoses), pneumonia (7%) and congestive heart failure (7%) (data not shown in tables).

Length of stay was greater in hospitalizations with substantial diagnostic discrepancy (median 5 days; interquartile range = 3-7 days) than in those without substantial diagnostic discrepancy (median 4 days; interquartile range = 2-5 days; P < 0.001), as was mortality (26 versus 12%; P < 0.001). Other characteristics that were found to be more frequent among patients with substantial diagnostic discrepancy included the following: residing in nursing homes, having altered levels of consciousness, presenting lower levels of mean arterial pressure and having more ED diagnostic and screening tests.

In a multivariate analysis model that included demographic and clinical covariates, we found that age, nursing home residence, altered level of consciousness, mean arterial pressure, number of ED diagnostic and screening tests and length of stay were independently associated with substantial diagnostic disagreement (Table 2). In addition, substantial diagnostic discrepancy was independently associated with in-hospital mortality (odds ratio = 2.2; P = 0.001), after adjusting for demographic and clinical covariates.

Because we excluded nonspecific diagnoses from consideration, our methods had the potential to misclassify certain patients as having substantial diagnostic discrepancy. For example, consider a patient with admission diagnosis #1 of urinary infection, admission diagnosis #2 of chest pain, and discharge diagnosis of acute myocardial infarction. In this case, the admission and discharge diagnoses would be classified as substantially discrepant because the nonspecific diagnosis “chest pain” would not be used for the comparison, and the remaining admission diagnosis (urinary infection) would be considered discrepant from the discharge diagnosis of acute myocardial infarction. We thus performed a conservative sensitivity analysis including only the records in which all of the admission diagnoses, as well as the discharge diagnosis, were specific. In this sensitivity approach, 5% of the patients aged 18-64 years had substantial diagnostic discrepancy versus 9% for those aged 65 years or more (P < 0.001).

## DISCUSSION

One out of eight older adults hospitalized from EDs in the United States with a specific admission diagnosis was discharged with a substantively different principal diagnosis. Diagnostic discrepancies were more common among older adults, occurring in 12.5% of adults over the age of 65 years, with a particularly high rate among patients aged 85 years or more (14.5%), while appearing in only 8.3% of those under 65 years. Moreover, substantial diagnostic discrepancy was independently associated with longer hospital stays and increased in-hospital mortality. Presenting clinical diagnosis, nursing home residence, lower mean arterial pressure and more ED diagnostic and screening tests were also independently associated with substantial diagnostic discrepancy.

Missed, delayed or incorrect diagnoses are estimated to occur in 5% to 20% of medical encounters.^18^^,^^19^ Older age is generally thought to be associated with diagnostic uncertainty and is therefore a risk factor for diagnostic errors, but the data reported so far have mostly been restricted to specific settings or clinical conditions such as malpractice claims, cancer, infections and myocardial infarction.^11^^,^^20^^-^^23^ In a study conducted in a university hospital, 230 patients (32%) admitted from the ED were discharged with diagnostic changes in relation to specificity and/or category, and this occurred more frequently among older patients.^16^ Another study that investigated ED admission-to-discharge discrepancies in an urban level-1 trauma hospital reported that the majority of the group with diagnostic disagreements comprised elderly people.^24^ To the best of our knowledge, ours was the first study to analyze this issue in a nationally representative sample of ED encounters and confirm that older age was associated with greater rates of admission-to-discharge diagnostic discrepancies.

The frequency of diagnostic discrepancies varied according to the presenting clinical diagnosis at admission. It is intriguing that substantial diagnostic discrepancy tended to be less likely to happen with the most frequent conditions, while less frequent illnesses were associated with its occurrence. Nevertheless, other studies have shown that a broad range of clinical conditions is frequently misdiagnosed, with similar patterns of individual and systemic vulnerabilities contributing to failures in diagnostic processes.^24^^,^^25^ Therefore, preventive measures that focus on addressing such systematic issues would likely have a more significant impact than those targeting specific diseases.

Discrepancies were also associated with factors potentially indicative of clinical severity, such as lower arterial blood pressure, altered level of consciousness and longer ED visits. It is plausible that patient characteristics contributing to clinical complexity increase the difficulty of establishing definite diagnoses. In a study on 307 closed malpractice claims alleging missed or delayed diagnosis in the ambulatory setting, Gandhi et al. reported that patient-related factors contributed to the errors in 46% of the cases.^25^ These included atypical clinical presentation and complicated medical history in 15% and 10% of the cases, respectively. However, most problems were also linked to cognitive factors relating to the care provider, such as clinical judgment and knowledge breakdowns.

Interestingly, substantial diagnostic discrepancy was also associated with the use of more diagnostic and screening tests in the ED. Although it is reasonable to infer that this is a consequence of greater clinical insecurity, complexity and severity in these cases, it is significant that these tests did not prevent substantial diagnostic discrepancy from happening after the admissions. This finding is consistent with previous reports suggesting that the rate of misdiagnosis has remained constant over the past decades notwithstanding all the technological advances that have been witnessed in the field of medicine.^24^^,^^26^ Accurate diagnosis still appears to be mostly dependent on the quality of patient histories and physical examinations.

Given the challenges that geriatric patients involve, it might be helpful to develop training programs to educate care providers regarding geriatric syndromes and possible atypical clinical presentations in this population. Specific policies for older adults allowing for more time for medical evaluations could also prove helpful.

Patients with diagnostic discrepancies had 25% longer hospital stays and more than twice the mortality. Johnson et al. found similar results in the general medicine units of a university hospital, and reported that patients admitted from the ED had 15% longer stays when disagreements were identified.^13^ It is important to note that not all diagnostic discrepancies in our study were necessarily due to misdiagnosis at the time of admission. Some of them may have been accounted for by new clinical conditions that arose during the hospitalization (i.e. nosocomial infections or new cardiovascular events, etc.), or even by poor documentation of critical diagnostic information. However, previous hospital-wide research has indicated that particularly high rates of diagnostic errors occur in the ED,^27^ and it is reasonable to assume that at least a proportion of the disagreements that we discovered were due to misdiagnosis. Lastly, a previous study that reviewed medical records in detail and analyzed discrepancies between primary ED admitting diagnosis and primary discharge diagnosis concluded that such inconsistencies could be used reliably to screen for missed diagnoses.^24^

In many cases, diagnoses can be elusive and wrong, even in the setting of good clinical care, especially when providing care for older adults who might have several concomitant chronic and acute conditions. We excluded from our analyses the cases that only had nonspecific admission and/or discharge diagnoses, in an attempt to minimize the effects of diagnostic uncertainty and diagnostic codes that would retrieve discrepancies merely due to lack of specificity. This strategy probably contributed towards underestimating the degree of diagnostic uncertainty and change that actually occurred. Conversely, aspects of our methods may have contributed towards overestimating the rate of diagnostic discrepancies through discounting any information available from nonspecific diagnoses, as explained in our sensitivity analysis. Although limitations to coding and to how the comparisons were defined made it difficult to establish a single precise “true” rate of diagnostic discrepancies, our main analyses and the sensitivity analysis were generally consistent. Hence, these analyses allowed us to: (1) provide information on the magnitude of diagnostic discrepancies among patients with at least one specific admission diagnosis; and (2) indicate that there was a large proportion of patients with non-specific admission diagnoses for whom diagnostic uncertainty and the possibility for discrepancies were even greater.

There were other limitations to this study, including its retrospective nature and imperfect measurement of potential confounders, along with the probability sample design of the NHAMCS dataset. Although the data collectors underwent training and the data were subject to a 10% random sample crosscheck, occurrences of residual errors in collection and coding cannot be ruled out. Specifically, regarding our study, the dataset had a restricted number of diagnostic variables and limited clinical information. Nonetheless, this dataset has strength as a nationally representative sample and has been widely used in similar analyses.^28^^-^^30^

## CONCLUSIONS

Missed and delayed diagnoses among older adults represent a critical patient safety problem, and diagnostic procedures are becoming increasingly complex and susceptible to failure.^25^ They are a leading cause of malpractice claims and preventable adverse events in hospitals, and frequently result from failure to hypothesize the correct diagnosis.^21^^,^^22^^,^^31^ Our results show that substantial admission-to-discharge diagnostic discrepancies occur more commonly among older adults hospitalized from the ED. These discrepancies may have occurred as a result of diagnostic error, coexistence of multiple conditions or development of new diseases. All of these possibilities have practical implications, and clinicians attending hospitalized older patients should be vigilant and consider alternative diagnostic possibilities early in the course of hospitalization. Future research should focus on interventions that might improve diagnostic practices for these patients, including geriatric training for in-hospital care providers and development of clinical decision support tools.
